# Residue analysis of a CTL epitope of SARS-CoV spike protein by IFN-gamma production and bioinformatics prediction

**DOI:** 10.1186/1471-2172-13-50

**Published:** 2012-09-10

**Authors:** Jun Huang, Yingnan Cao, Xianzhang Bu, Changyou Wu

**Affiliations:** 1 Institute of Immunology, Zhongshan School of Medicine; Key Laboratory of Tropical Disease Control Research of Ministry of Education, Sun Yat-sen University, Guangzhou, China; 2 School of Pharmaceutical Science, Sun Yat-sen University, Guangzhou, China; 3 Department of Pathogenic Biology and Immunology, Guangzhou Medical College, Guangzhou, China; 4 Department of Pharmacy, Xinhua College of Sun Yat-sen University, Guangzhou, China

**Keywords:** SARS-CoV, CTL, Epitope, Residue

## Abstract

**Background:**

Severe acute respiratory syndrome (SARS) is an emerging infectious disease caused by the novel coronavirus SARS-CoV. The T cell epitopes of the SARS CoV spike protein are well known, but no systematic evaluation of the functional and structural roles of each residue has been reported for these antigenic epitopes. Analysis of the functional importance of side-chains by mutational study may exaggerate the effect by imposing a structural disturbance or an unusual steric, electrostatic or hydrophobic interaction.

**Results:**

We demonstrated that N50 could induce significant IFN-gamma response from SARS-CoV S DNA immunized mice splenocytes by the means of ELISA, ELISPOT and FACS. Moreover, S366-374 was predicted to be an optimal epitope by bioinformatics tools: ANN, SMM, ARB and BIMAS, and confirmed by IFN-gamma response induced by a series of S358-374-derived peptides. Furthermore, each of S366-374 was replaced by alanine (A), lysine (K) or aspartic acid (D), respectively. ANN was used to estimate the binding affinity of single S366-374 mutants to H-2 Kd. Y367 and L374 were predicated to possess the most important role in peptide binding. Additionally, these one residue mutated peptides were synthesized, and IFN-gamma production induced by G368, V369, A371, T372 and K373 mutated S366-374 were decreased obviously.

**Conclusions:**

We demonstrated that S366-374 is an optimal H-2 Kd CTL epitope in the SARS CoV S protein. Moreover, Y367, S370, and L374 are anchors in the epitope, while C366, G368, V369, A371, T372, and K373 may directly interact with TCR on the surface of CD8-T cells.

## Background

Severe acute respiratory syndrome (SARS) is an emerging infectious disease caused by the novel coronavirus SARS-CoV [1,2]. The fatality rate is as high as 15% for patients younger than 60 years old and can be higher than 50% for patients 60 years or older. Nearly 40% of infected patients develop respiratory failure that requires assistant ventilation [3].

Coronaviruses (CoVs) are positive-strand RNA viruses. The virion consists of a nucleocapsid (N) core surrounded by an envelope containing three membrane proteins, spike (S), membrane (M) and envelope (E), which are common to all members of the genus [4,5]. The M and E proteins are important for viral particle assembly and N is important for viral RNA packaging. The S protein, which provides the virion with a corona-like appearance, binds to host receptors and mediates membrane fusion [6]. The successful development of effective treatments and vaccines against SARS-CoV depends on understanding the roles of various immune effectors in protective immunity and on identifying protective viral antigens recognized by these effector cells. In a preliminary study, the S protein fragment S358–374 was shown to stimulate the production of IFN-γ by CD8-T cells from immunized BALB/c mice [7].

The capacity of a diverse array of peptides to bind to an individual class I molecule is due to anchor residues in the peptides [8]. The surface features of the binding cleft of the class I MHC molecule are complementary to side chains of the anchor residues in the displayed peptide. The amino acid residues lining the binding sites may vary among different class I allelic variants [9,10]. Here, an SARS CoV S protein CTL epitope, S366–374, was identified and the functions of individual residues were evaluated by bioinformatics tool prediction and by IFN-γ responses induced by a series of modified S366–374 peptides.

## Methods

### Mice

Female BALB/c mice, 6–8 weeks old, were purchased from Zhongshan University Animal Center (Guangzhou, China) and maintained in our animal care facility under pathogen free conditions. For experimental purposes, six to eight week-old female mice were used. All experiments were performed according to the guidelines in the Institutional Animal Committee of Zhongshan School of Medicine, China.

### SARS-CoV S DNA vaccine

Plasmids encoding SARS-CoV spike (S) protein was constructed as described [11], and kindly provided by Dr. Gary J. Nabel from Vaccine Research Center, NIAID, National Institutes of Health, MD, USA. Plasmid DNA was purified by plasmid-purified kit (QIAGEN, USA). The 260/280 ratios ranged from 1.8 to 2.0. The endotoxin content from purified plasmid DNA was found below 20 U/ml. The endotoxin level within this range had no effect on the immune response.

### Synthesis, purification and analysis of S358-374 analogs

The peptides were synthesized by solid phase using an Fmoc strategy [12]. 2-Chlorotrityl chloride resin loaded at 1.0 mmol/g (GL BIO Company, Shanghai, China) which was used in peptide synthesis and was chosen as the solid support. N-protected Fmoc amino acids were used. For functionalized amino acids, the following derivatives were used: Fmoc-Cys(trt)-OH, Fmoc-Leu-OH, Fmoc-Val-OH, Fmoc-Gly-OH, Fmoc-Ala-OH, Fmoc-Ser(tBu)-OH, Fmoc-Thr(tBu)-OH, Fmoc-Tyr(tBu)-OH, and Fmoc-Lys(Boc)-OH and were from GL BIO Company (Shanghai, China). The coupling reagent was 2-(1 H-benzotriazol-1-yl)-1,1,3,3-tetramethyluronium hexafluorophosphate for all peptides. Syntheses of the different peptides were performed from 0.07 g of 2-Chlorotrityl chloride resin. An excess of 8 eq of each amino acid was used.

Fmoc deprotection was performed with a solution of piperidine in dimethylformamide in a 2/8 (v/v) ratio. Final deprotection of the peptides from the resin was performed in a mixture containing trifluoroacetic acid, phenol, water, triisprpylsilane in a 88/5/5/2 (v/v) ratio for 3.5 h. Peptides were then precipitated by addition of cold diethyl ether and dissolved in a mixture of 0.1% trifluoroacetic acid in water/acetonitrile and lyophilized. Purity of all peptides was checked by analytical high performance liquid chromatography on a Waters instrument using a C18 column (Novarpack, 5 μm, 300 Å, 10.0 × 200 mm) and all were at least 70% pure (UV detection at 214 and 254 nm). They were characterized by electrospray mass spectrometry.

### Immunization of mice

Female BALB/c mice were injected (i.m.) with 50 μg/mouse of SARS-CoV S plasmid DNA in 100 ul of sterile PBS. Mice were boosted twice at 2–3 weeks interval.

### Cell culture and IFN-γ ELISA

Mice were sacrificed. Spleen from individual mouse was harvested one to two weeks after the final boost vaccination. Single cell suspensions were prepared and plated in a 96-well micro-titer plate at 4 × 10^5^ cells/200 μl per well. Pooled SARS CoV S peptides (1 μg/ml for each) or single peptide (1 μg/ml) with anti-mouse CD28 mAb (1 μg/ml) were added to cultures. Supernatants of cell cultures were collected 72 h later, and levels of IFN-γ were assessed by specific ELISA kit (BD PharMingen) according to the manufacturer’s protocol. The detection limit of the IFN-γ assay kit was 3.13 pg/ml.

### IFN-γ ELISPOT

Assessment of SARS-CoV S-specific IFN-γ producing cells after vaccination was determined by ELISPOT (Diaclone, France) according to the manufacturer’s protocol. In brief, single cell suspensions were prepared from spleens of mice after vaccination, and plated in 96-well microplate precoated with anti-IFN-γ antibody specific for ELISPOT. Cells were incubated overnight in the presence or absence of peptide (1 μg/ml) and anti-CD28 (1 μg/ml). The plates were then washed and alkaline phosphatase conjugated anti-mouse IFN-γ antibody was added, developed with ready-to-use BCIP/NBT, and read by Champ Spot II ELISPOT reader (Sage Creation, China).

### Cell surface and intracellular cytokine staining

Single-cell suspensions from spleens of mice after vaccination were stimulated with or without SARS CoV S peptides plus anti-CD28 (1 μg/ml) for 5 h at 37°C and 5% CO_2_. Brefeldin A (10 μg/ml, Sigma) was added in the last 4 h incubation. Cells were washed, fixed with 4% paraformaldehyde and permeabilized in PBS buffer containing 0.1% saponin (Sigma), 0.1% BSA and 0.05% NaN_3_ overnight at 4°C. Cells were then stained with conjugated mAbs specific for CD4, CD8 and intracellular cytokine IFN-γ for 20–3 0 min at 4°C in dark. Cells (300,000) were acquired on flow cytometer (BD Calibur) and data were analyzed with FlowJo program, version 6.0 (Tree Star, Inc., USA). Isotype matched controls for cytokines were included in each staining.

### Bioinformatics analysis

T cell epitope prediction tools, ANN, SMM and ARB provided by IEDB are publicly available in a website (http://tools.immuneepitope.org/analyze/html/mhc_binding.html) Version 2009-09-01B.

Artificial Neural Network (ANN) is a connectionist models that consist of a number of interconnected units that can be activated by transmitting signals [13,14]. ANN can tolerate a degree of erroneous data, and can classify nonlinear data, which makes them highly suitable for processing noisy biological information. ANN applications have been described for predictions of MHC class I binding peptides and for MHC class II peptides. The prediction accuracy of ANN-based methods was reported to be close to 80% sensitivity and 80% specificity [15].

Stabilized Matrix Method (SMM) is a T cell epitope predictive tool based on quantitative matrices. It has been successfully applied to predicting peptide binding to MHC molecules, peptide transport by the transporter associated with antigen presentation (TAP) and proteasomal cleavage of protein sequences [16,17]. The sensitivity of SMM-based methods was reported to be close to 60% [18].

Average Relative Binding (ARB) is a matrix method, which allows combination of searches involving different peptide sizes and alleles into a single global prediction [19]. ARB has achieved a favorable performance in predicting MHC I and MHC II molecules [20,21].

Application of these tools was according to prompt dialog box showed in the website. In brief: a) Choose MHC class I model. b) Enter sequence of various peptides. c) Choose prediction method as ANN, SMM and ARB, respectively. d) Specify what to make binding predictions for. Select MHC source species as mouse, allele as H-2K^d^, length as 9. e) Specify output. These tools predict IC_50_ values for peptide binding to specific MHC molecules. IC_50_ value means binding ability. Note that binding to MHC is necessary but not sufficient for recognition by T cells.

HLA Peptide Binding Predictions tool provided by BioInformatics and Molecular Analysis Section (BIMAS) (http://www-bimas.cit.nih.gov/cgi-bin/molbio/ken_parker_comboform) was used to compare the binding kinetics of peptides. The analysis is based on coefficient tables deduced from the published literature by Dr. Kenneth Parker [22]. Application of this tool was according to prompt dialog box provided by the website. Higher half lime value means better binding ability.

### Statistics

Statistical evaluation of differences between means of experimental groups was performed by analysis of variance and a non-parametric two-tailed *t* test. P value <0.05 was considered to be significant.

## Results

### N50 is a MHC-I restricted peptide in SARS-CoV S protein

**Figure 1:**
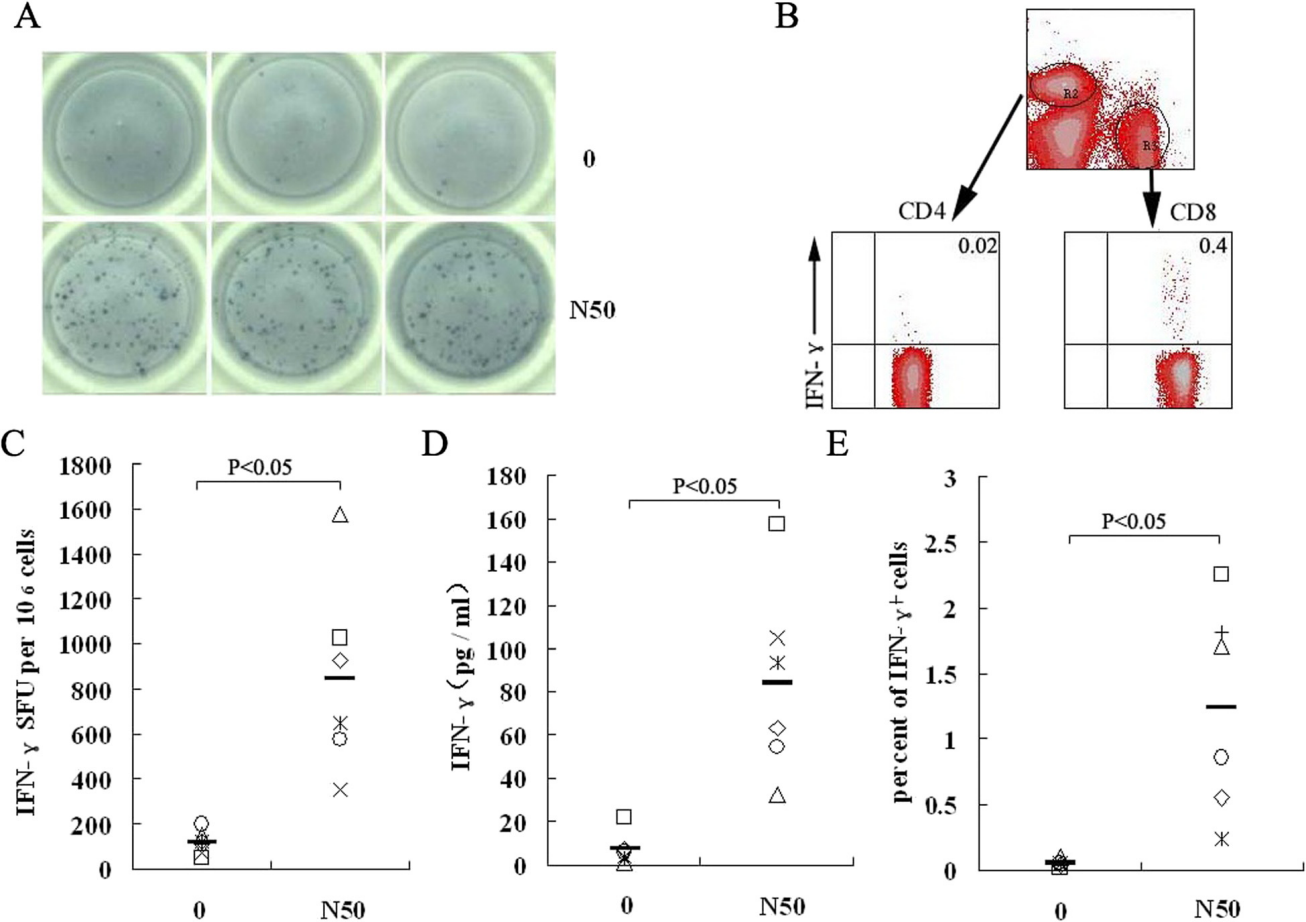
**The production of IFN-γ induced by peptide N50.** BALB/c mice were immunized (i.m.) by SARS CoV S DNA. One to two weeks after the final boost immunization, splenocytes were prepared and stimulated with peptide N50. (**A**) After 14–18 h, the number of IFN-γ producing cells was detected by ELISPOT. Representation of ELISPOT results was shown. (**B**) After 4–5 h, FACS was performed to determine the percentages of IFN-γ^+^ cells in both CD4^+^ and CD8^+^ T cell population. Representation of FACS results was shown. Numbers at the corner in each sample represent the percentage of positive cells. (**C**) ELISPOT results. (**D**) After 72 h, supernatants were collected and levels of IFN-γ were detected by ELISA. (**E**) FACS results. Each open symbol represents mean value of an independent experiment (n =6). Cross bar represents the mean result. “0” represent non-peptide control.

To identify SARS CoV S epitopes, the potential SARS-CoV S epitopes were tested repeatedly by splenocytes from DNA vaccine immunized BALB/c mice. ELISA and ELISPOT results indicated that the adjacent peptides P50 and P51 possessed the same ability to induce IFN-γ production [9]. The overlapping sequence between P50 and P51 (N50, KCYGVSATKL) was synthesized. ELISA (Figure 1A), ELISPOT (Figure 1B/D) and FACS results indicated that peptide N50 could induce IFN-γ production. The FACS results showed that N50 could only induced CD8^+^ T cells to produce IFN-γ (Figure 1C/E).

### Amino acid residue L^374^ is essential for stimulation of IFN-γ production in response to S365-374

**Figure 2:**
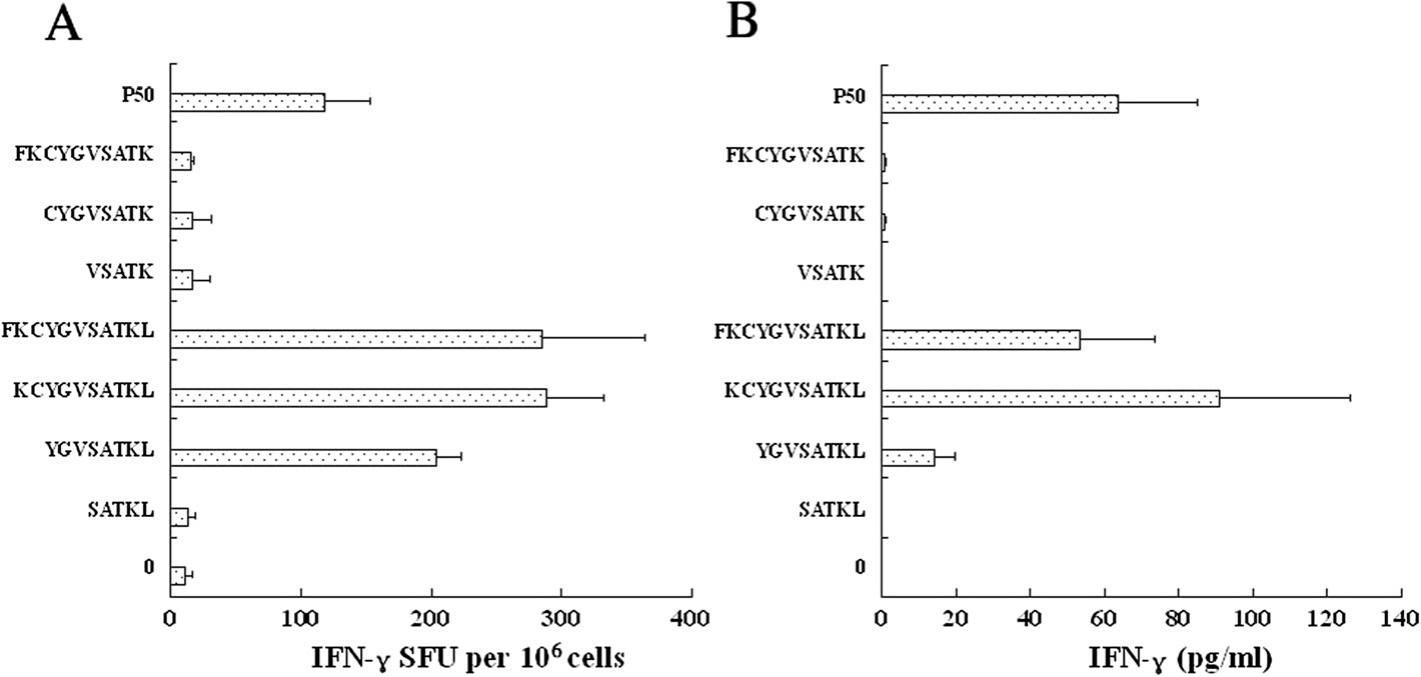
**IFN-γ induced by seven S366–374-derived peptides.** BALB/c mice were immunized by SARS CoV S DNA vaccine. Splenocytes were prepared and stimulated with seven peptides derived from S366–374 (KCYGVSATKL). ELISPOT (**A**) and ELISA (**B**) were performed to detect the production of IFN-γ. “0” represent unstimulated controls. Experiments were performed in duplicate and representative results are shown.

To identify the optimal epitope in S365–374, a series of S358–374-derived peptides were synthesized and used to stimulate splenocytes from SARS-CoV S DNA vaccine immunized BALB/c mice. The fraction of IFN-γ-producing T cells was determined by ELISPOT (Figure 2A), and the level of IFN-γ in supernatants was measured by ELISA (Figure 2B). Both results indicated that IFN-γ was produced only in response to peptides preserving residue L^374^. Thus, S367–374 (YGVSATKL), S365–374 (KCYGVSATKL), and S364–374 (FKCYGVSATKL) could elicit robust IFN-γ production. Only S370–374 (SATKL) was inactive, likely due to weak affinity to MHC-I (data not shown). In contrast, L^374^ deleted peptides, including S369–373 (VSATK), S366–373 (CYGVSATK), and S363–373 (FKCYGVSATK) could not induce IFN-γ production. The IFN-γ response induced by S365–374 was much stronger than that induced by S367–374 (P < 0.05).

### S366-374 is the optimal epitope

To identify the optimal epitope, we analyzed the binding affinity of S365–374 peptides to H-2 K^d^, H-2 D^d^, and L^d^ by several bioinformatics tools. The MHC-binding scores were determined by three peptide-binding prediction methods: artificial neural network (ANN) [23], stabilized matrix method (SMM) [16], and average relative binding (ARB) [19]. Predicted binding scores were expressed as IC_50_ values, which represented the equilibrium dissociation constant (KD) of the peptide in relation to a particular MHC molecule. The binding affinities of all 9 and 10 amino acid peptide stretches in S358–381 were predicted. The data indicated that the binding of 9 aa peptides was stronger than all 10 aa peptides and that these 9 aa peptides binded with higher affinity to H-2 K^d^ than to H-2 D^d^ or H-2 L^d^ (data not shown). Therefore, we concluded that the optimal epitope should be an H-2 K^d^ restricted 9 amino acids peptide. In addition, the results demonstrated that S366–374 (CYGVSATKL) was the highest affinity peptide to H-2 K^d^ (Table 1).

**Table 1:** Predicted MHC-peptide binding

**Sequence**	**ANN**	**SMM**	**ARB**
STFFSTFKC	38169.9	44479.5	16214.6
TFFSTFKCY	34824.9	468823.1	191908.2
FFSTFKCYG	36550.6	165788.7	202312.4
FSTFKCYGV	37493.9	270563	24611.6
STFKCYGVS	37025.3	36095.8	11060.5
TFKCYGVSA	36950.6	857351.7	1000000
FKCYGVSAT	28781	9145.4	180.1
KCYGVSATK	36702.2	4243.3	476309.3
CYGVSATKL	59.2	84.1	3.7
YGVSATKLN	39136.2	114095.1	1000000
GVSATKLND	39141.7	224004.7	1000000
VSATKLNDL	26880.9	23063.8	59717.3
SATKLNDLC	35465.1	10192.3	206549.5
ATKLNDLCF	39187.4	4083163	1000000
TKLNDLCFS	38468.4	807152.2	357259.1
KLNDLCFSN	37970.1	12958.7	203755.1

The binding affinities of 9 aa peptides in S358–382. (STFFSTFKCYGVSATKLNDLCFSN) to H-2 K^d^ were predicted by computer algorithms. Numbers in the table are values of IC_50_ (nM) determined by ANN, SMM, and ARB. IC_50_ = 50% inhibitory concentration (low IC_50_ values indicate high affinity binding).

The epitope mapping tool BIMAS [22] (http://www-bimas.cit.nih.gov) was used to compare the binding kinetics of three peptides containing L^374^ as the N-terminal peptide (S366–374, S365–374, and S367–374). The half time of disassociation from H-2 K^d^ of each these molecules was estimated. The score for S366–374 (CYGVSATKL) was 2880, much higher than for S365–374 and S367–374 (Table 2).

**Table 2:** Estimated half time of disassociation of peptides

**Sequence**	**Score**
YGVSATKL	96
CYGVSATKL	2880
KCYGVSATKL	69.12

Score means Estimate of Half Time of Disassociation of the Subsequence.

### Predicted binding affinity of the S366-374 series with one mutated residue

To elicit an effective IFN-γ response, an epitope must bind to an MHC molecule first. Artificial neural network (ANN) was used to estimate the binding affinity of single S366–374 mutants to H-2 K^d^ (Table 3). The IC_50_ value of wild type S366–374 (CYGVSATKL) was 59.2 nM. When Y^367^ and L^374^ were replaced by alanine (A), lysine (K), or aspartic acid (D), the IC_50_ value increased dramatically (to more than 28,000 nM), indicating that Y^367^ and L^374^ were important for peptide binding and might serve as the main anchors in this epitope.

**Table 3:** Predicted MHC-I binding affinities of A-, K-, and D-substituted S366–374 peptides

	**Sequence**	**IC50 (nM)**		**Sequence**	**IC50 (nM)**		**Sequence**	**IC50 (nM)**
C^366^A	AYGVSATKL	66	C^366^K	KYGVSATKL	15.5	C^366^D	DYGVSATKL	551.5
Y^367^A	CAGVSATKL	25435	Y^367^K	CKGVSATKL	22549	Y^367^ D	CDGVSATKL	28232
G^368^A	CYAVSATKL	7.3	G^368^K	CYKVSATKL	18.5	G^368^ D	CYDVSATKL	150
V^369^A	CYGASATKL	13.8	V^369^K	CYGKSATKL	69.6	V^369^ D	CYGDSATKL	791.1
S^370^A	CYGVAATKL	216.1	S^370^K	CYGVKATKL	1118	S^370^ D	CYGVDATKL	959.9
	CYGVSATKL	59.2	A^371^K	CYGVSKTKL	40.3	A^371^ D	CYGVSDTKL	112.9
T^372^A	CYGVSAAKL	1995	T^372^K	CYGVSAKKL	3122	T^372^ D	CYGVSADKL	882.4
K^373^A	CYGVSATAL	13.3		CYGVSATKL	59.2	K^373^ D	CYGVSATDL	38.5
L^374^A	CYGVSATKA	9265	L^374^K	CYGVSATKK	22172	L^374^ D	CYGVSATKD	25522

Numbers in the table are values of IC_50_ (nM) predicted by artificial neural network (ANN).

**Figure 3:**
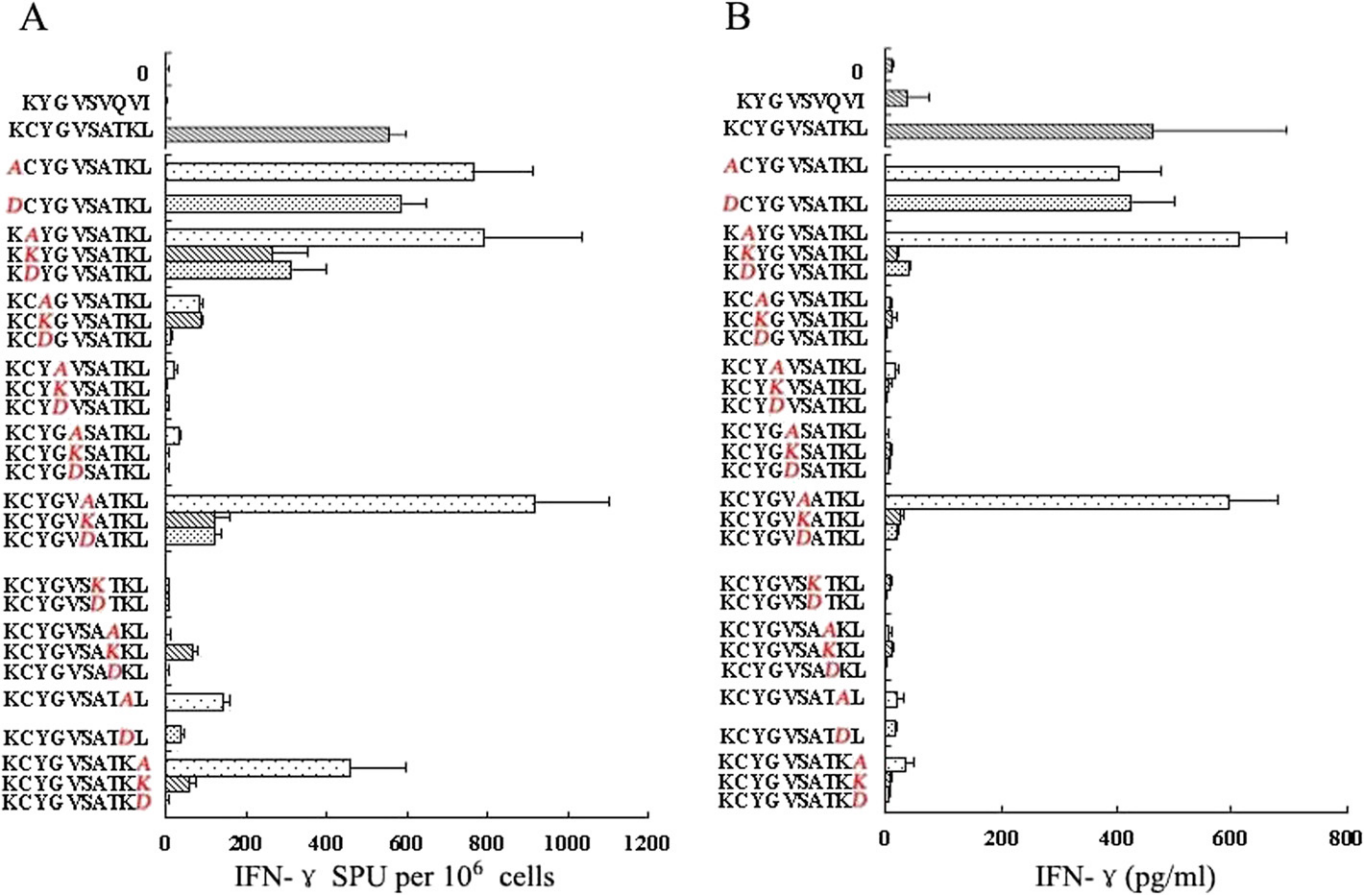
**The production of IFN-γ induced by mutated S366–374.** BALB/c mice were immunized by SARS CoV S DNA vaccine and splenocytes were prepared as described previously [7]. Each amino acid residue in S366–374 was replaced by alanine (A), lysine (K), or aspartic acid (D). The 27 distinct S366–374 mutants were used to stimulate splenocytes. (**A**) After incubation for 14–18 h, the frequency of IFN-γ spot forming unit (SFU) was detected by ELISPOT. (**B**) After incubation for 72 h, supernatants were collected and levels of IFN-γ were detected by ELISA. “0” represent unstimulated control. KYGVSATKL was a scrambled peptide control. Experiments were performed in duplicate and representative results are shown.

Although G^368^A (G^368^ replaced by A) and G^368^K isoforms of S366–374 possessed higher affinity than wild type S366–374, they could not elicit IFN-γ responses (Figure 3), indicating that G^368^ might directly contact the T-cell receptor. The residues V^369^, A^371^, and K^373^ had functions similar to G^368^, while the role of S^370^ was distinct. The IC_50_ value of S^370^A was 216.5 nM, about 4 times higher than wild type S366–374. The IC_50_ values of S^370^K and S^370^D were nearly 1000 nM, indicating that S^370^ might be a weak anchor to H-2 K^d^. The role of T^372^ was similar to S^370^, as the IC_50_ values of T^372^A, T^372^K, and T^372^D ranged from nearly 1000 nM to 3000 nM.

### The production of IFN-γ induced by one residue-mutated S366-374

To confirm the key residues in S366–374, 27 analogs were synthesized in which each of the nine residues was replaced by A, K, or D. These peptides were used to stimulate splenocytes from SARS CoV S DNA immunized mice. ELISPOT (Figure 3A) and ELISA (Figure 3B) were performed to detect IFN-γ production.

The frequency of IFN-γ producing cells induced by K^365^A or K^365^D was similar to that induced by wild type S365–374 (KCYGVSATKL), and there was no obvious difference in the IFN-γ levels in the culture cell supernatants following peptide treatment. When C^366^ was replaced by A, the frequency of IFN-γ producing cells and the IFN-γ levels in the supernatant were also similar to wild type S365–374. When C^366^ was replaced by K or D, the frequency of IFN-γ producing cells was decreased by nearly 50%, and the level of IFN-γ in the supernatant was much lower than that induced by wild type S365–374. The S^370^ residue appeared to be more important than C^366^ for induction of IFN-γ as IFN-γ responses induced by S^370^A were similar to that induced by wild type S365–374, while treatment with S^370^K and S^370^D peptides resulted in a 75% lower frequency of IFN-γ producing cells and a significantly reduced supernatant IFN-γ concentration compared to wild type S365–374.

The L^374^ was also an essential residue in the epitope. No IFN-γ response was detected in mutated peptides without L^374^ (Figure 2). However, when L^374^ was replaced by A, IFN-γ responses could still be detected. The frequency of IFN-γ producing cells following L^374^A treatment was about half that induced by wild type S365–374. When L^374^ was replaced by K, some IFN-γ spot forming units (SFUs) could also be detected. In addition, Y^367^ was important for IFN-γ induction; although about 200 SPU per 10^6^ cells could be induced by Y^367^A or Y^367^K, the level of IFN-γ in the supernatant was significantly reduced compared to that induced by wild type S365–374. The K^373^ residue had a role similar to Y^367^. When K^373^ was replaced by A or D, the IFN-γ responses were similar to those induced by Y^367^ mutated peptides.

The most important residues in the epitope were G^368^, V^369^, A^371^, and T^372^. Almost no IFN-γ response could be detected when these residues were replaced by A, K, or D.

### C^366^A and S^370^A are H-2 K^d^ restricted epitopes

**Figure 4:**
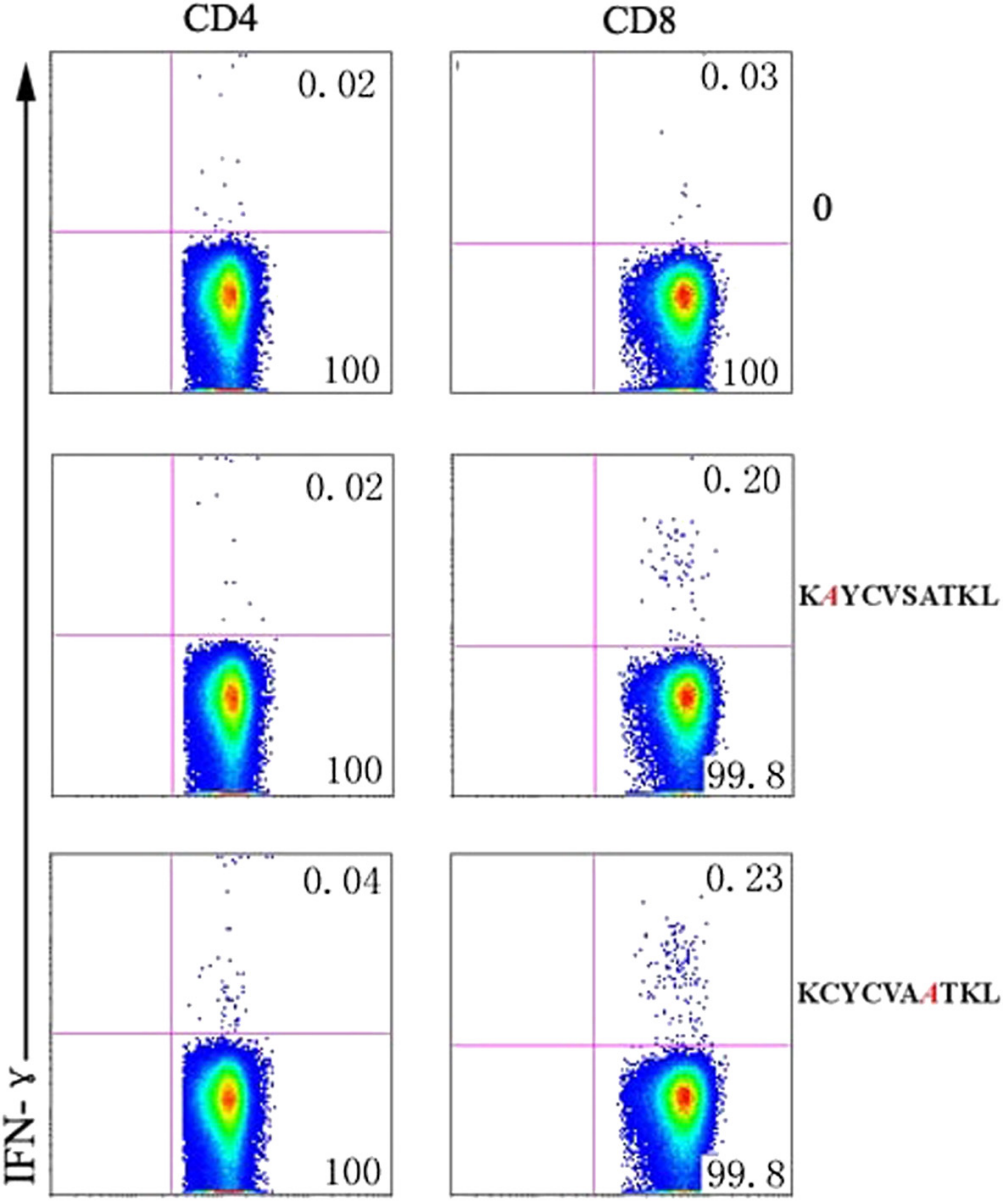
**KAYGVSATKL and KCYGVAATKL could induce CD8**^+^**T cells produce IFN-γ.** BALB/c mice were immunized as described previously [7]. Splenocytes were prepared and stimulated with KAYGVSATKL and KCYGVAATKL. FACS was performed to determine the expression of IFN-γ in CD4^+^ and CD8^+^ T cells. Numbers at the corner represent the percentage of positive (expressing) cells. Representative results from three independent experiments are shown.

As shown in Figure 4, IFN-γ responses induced by C^366^A and S^370^A were similar to that elicited by wild type S365–374, indicating that C^366^ and S^370^ could be replaced by A without affecting peptide function. To further confirm this result, fluorescence activated cell sorting (FACS) was performed. The result showed that C^366^A and S^370^A could only stimulate CD8^+^ T cells to produce IFN-γ, indicating that both were H-2 K^d^ restricted epitopes.

## Discussion

The T cell epitopes of the SARS CoV spike protein are well known, but systematic evaluation of the functional and structural roles of each residue has not been reported for these antigenic epitopes [24-26]. In the preliminary study [7], by using a synthesized peptide pool of SARS CoV S protein to stimulate the splenocytes from SARS CoV S DNA vaccine immunized mice, we identified that the peptides of P50 and P51 could induce IFN-γ responses. P50 and P51 contained a same animo sequence, N50 (S365-374, KCYGVSATKL). In present study, N50 was synthesized and used to stimulate the splenocytes from immunized mice, ELISA, ELISPOT and FACS results all indicated that N50 contained a main CD8^+^ T cell epitope (Figure 1). Moreover, S366–374 (CYGVSATKL) was shown to be an optimal H-2 K^d^ restricted epitope of the SARS CoV S protein by both bioinformatics prediction (Tables 1 and 2) and a functional INF-γ release assay (Figure 2, Figure 3).

To induce effective T cell responses, the T cell epitope must bind to an MHC molecule. All peptides that bind to class I molecules contain a carboxyl-terminal anchor [27,28]. The anchor residues at both ends of the peptide are buried within the binding cleft, holding the peptide firmly in place. A previous study demonstrated that nonameric peptides bound preferentially and that the main contacts between class I MHC molecules were residue 2 at the amino-terminal end and residue 9 at the carboxyl terminus of the nonameric peptide. These anchors were generally hydrophobic residues (e.g., leucine and isoleucine) [29].

Site-directed mutagenesis is a powerful tool for probing protein or peptide structure and function. Alanine-scanning, lysine-scanning, or aspartic acid-scanning by systematic replacement of side-chains with alanine, lysine, or aspartic acid have been used widely to study binding sites on proteins [22,30]. Analysis of the functional importance of side-chains by mutational study may exaggerate the effect by imposing a structural disturbance or an unusual steric, electrostatic, or hydrophobic interaction. Alanine-scanning is the least disruptive to the peptide structure because alanine is uncharged and has the smallest amino acid side group next to glycine, and thus has been particularly useful for mapping protein functional domains. In contrast, glycine can change the main chain conformation of the protein [31], so individual alanine mutations are preferred to infer the roles of individual amino acid residues. Charged residues such as lysine and aspartic acid are capable of forming ion pairs and hydrogen bonds, so they often play an important role in protein binding as well as in the recognition of interacting proteins. Thus, lysine and aspartic acid are often used as substitutes to study electrostatic effects between proteins [32].

Computational prediction and modeling of MHC/peptide binding can greatly facilitate peptide screening, with tremendous savings in time and experimental effort. Using these methods, T cell epitopes in many vaccine candidates were identified [33,34]. A number of prediction servers are available for identification of peptides that bind MHC molecules. Lin et al. have reported a comparative evaluation of thirty prediction servers for seven human MHC-I molecules. It showed that the best prediction server across all HLA molecules in this study is NETM_ANN, closely followed by IEDB ANN and IEDB SMM [35,36].

In this study, both peptide-binding prediction methods and functional experiments were used to evaluate the roles of the different residues within the optimal epitope S365–374. Though K^365^ was excluded from this optimal epitope (Tables 1 and 2), the wild type S365–374 containing K^365^ induced a strong IFN-γ response (Figure 2). Thus, K^365^ was included in these synthesized mutant peptides. A comparison of IFN-γ responses showed that mutants K^365^A or K^365^D were as potent as wild type S365–374 (Figure 3), demonstrating that K^365^ is not in this optimal epitope and that residue in this position do not influence the function of S366–374.

The L^374^ at position P9 was predicted to be the carboxyl-terminal anchor in the epitope by ANN (Table 3) and the functional experiment confirmed that analogs without L^374^ could not induce IFN-γ secretion from immunized mouse splenocytes (Figure 2). However, L^374^A could still induce significant IFN-γ secretion, while L^374^K and L^374^D could not, suggesting that electrostatic effects and hydrophobic interaction may play an important role at this position.

Residue 2 at the amino-terminal end is another important anchor in many epitopes. In S366–374, Y^367^ at position P2 was predicted to be another important anchor by ANN (Table 3). The functional assay showed that Y^367^ mutated peptides (Y^367^A and Y^367^K) could induce IFN-γ responses, but that IFN-γ production was lower than that induced by S366–374. That might indicate an important role for the Y side chain in determining the binding affinity to H-2 K^d^[10].

The S^370^ at P5 is also a functionally significant residue in this epitope. Though not a traditional anchor, bioinformatics tools indicated that it might act as a weak anchor for H-2 K^d^ binding. The functional assay indicated that IFN-γ responses induced by S^370^ mutant epitopes were stronger than L^374^ mutant epitopes.

X-ray crystal lographic analyses of peptide-class I MHC complexes has revealed how the peptide-binding cleft in a given MHC molecule interacts stably with peptides [37]. Vesselin Mitaksov described the crystal structure of the MHC class I protein H2-K^d^ in complex with the antigenic peptide TYQRTRALV (Flu) derived from an influenza nucleoprotein. They found that Flu residues Tyr ^P2^, Thr^P5^, and Val^P9^ were sequestered into the B, C, and F pockets of the K^d^ groove, respectively [32]. In the sequence of S366–374, the positions Tyr^367^, Ser^370^ and Leu^374^ were at P2, P5, and P9 as well, implying that Tyr^367^, Ser^370^ and Leu^374^ are anchors in this optimal CTL epitope.

All three bioinformatics tools indicated that C^366^ was included in this optimal epitope (Table 1). In its absences, however, S367–374 could still induce an IFN-γ response, albeit weaker than that induced by wild type S365–374 (Figure 2). Thus, C^366^ could influence the function of this epitope. The ANN tool predicated that C^366^ was not an anchor to H-2K^d^ and C^366^ mutant peptides could still induce strong IFN-γ responses, especially C^366^A, so our results indicate that C^366^ is not an important residue for the peptide-TCR interaction. Conversely, mutations of G^368^, V^369^, A^371^, T^372^, and K^373^ induced only modest IFN-γ production, demonstrating that these residues made greater contributions in presenting to TCR in this optimal CTL epitope. As predicated by ANN, T^372^ contributed to H-2 K^d^ binding (Table 3). Indeed, IFN-γ responses induced by T^372^ mutant peptides were significantly weaker than peptides containing T^372^, indicating that T^372^ might contribute to the TCR interaction.

## Conclusions

In the present study, we demonstrate that S366–374 is an optimal H-2 K^d^ CTL epitope in the SARS CoV S protein. Moreover, Y^367^, S^370^, and L^374^ are anchors in the epitope, while C^366^, G^368^, V^369^, A^371^, T^372^, and K^373^ may directly interact with TCR on the surface of CD8-T cells.

## Abbreviations

SARS: Severe acute respiratory syndrome; CoV: Coronavirus; S: Spike protein; SFU: Spot forming unit; FACS: Fluorescence activated cell sorter; CTL: Cytotoxic lymphocyte; BFA: Brefeldin A; ANN: Artificial neural network; SMM: Stabilized matrix method; ARB: Average relative binding.

## Competing interests

The authors declare that they have no competing interests.

## Authors’ contributions

JH carried out the immunoassays and helped to draft the manuscript. YC JH carried out peptide synthesis, purification and analysis. XB participated in its design and coordination and helped to draft the manuscript. CW conceived of the study, and participated in its design and coordination and helped to draft the manuscript. All authors read and approved the final manuscript.

## References

[B1] Drosten C Gunther S Preiser W van der Werf S Brodt HR Becker S Rabenau H Panning M Kolesnikova L Fouchier RA Identification of a novel coronavirus in patients with severe acute respiratory syndrome N Engl J Med 2003 348 1967 1976 10.1056/NEJMoa030747 12690091

[B2] Peiris JS Lai ST Poon LL Guan Y Yam LY Lim W Nicholls J Yee WK Yan WW Cheung MT Coronavirus as a possible cause of severe acute respiratory syndrome Lancet 2003 361 1319 1325 10.1016/S0140-6736(03)13077-2 12711465 PMC7112372

[B3] Tse GM To KF Chan PK Lo AW Ng KC Wu A Lee N Wong HC Mak SM Chan KF Pulmonary pathological features in coronavirus associated severe acute respiratory syndrome (SARS) J Clin Pathol 2004 57 260 265 10.1136/jcp.2003.013276 14990596 PMC1770245

[B4] Rota PA Oberste MS Monroe SS Nix WA Campagnoli R Icenogle JP Penaranda S Bankamp B Maher K Chen MH Characterization of a novel coronavirus associated with severe acute respiratory syndrome Sci 2003 300 1394 1399 10.1126/science.1085952 12730500

[B5] Marra MA Jones SJ Astell CR Holt RA Brooks-Wilson A Butterfield YS Khattra J Asano JK Barber SA Chan SY The genome sequence of the SARS-associated coronavirus Sci 2003 300 1399 1404 10.1126/science.1085953 12730501

[B6] He Y Zhou Y Siddiqui P Jiang S Inactivated SARS-CoV vaccine elicits high titers of spike protein-specific antibodies that block receptor binding and virus entry Biochem Biophys Res Commun 2004 325 445 452 10.1016/j.bbrc.2004.10.052 15530413 PMC7092874

[B7] Huang J Cao Y Du J Bu X Ma R Wu C Priming with SARS CoV S DNA and boosting with SARS CoV S epitopes specific for CD4+ and CD8+ T cells promote cellular immune responses Vaccin 2007 25 6981 6991 10.1016/j.vaccine.2007.06.047 PMC7115420 17709158

[B8] Laugel B van der Berg HA Gostick E Cole DK Wooldridge L Boulter J Milicic A Price DA Sewell AK Different T cell receptor affinity thresholds and CD8 coreceptor dependence govern cytotoxic T lymphocyte activation and tetramer binding properties J Biol Chem 2007 282 23799 23810 10.1074/jbc.M700976200 17540778

[B9] Anderson MW Gorski J Cutting edge: TCR contacts as anchors: effects on affinity and HLA-DM stability J Immunol 2003 171 5683 5687 14634075 10.4049/jimmunol.171.11.5683

[B10] Lee Y Ferrari G Lee SC Estimating design space available for polyepitopes through consideration of major histocompatibility complex binding motifs Biomed Microdevices 2010 12 207 222 10.1007/s10544-009-9376-7 20033850

[B11] Yang ZY Kong WP Huang Y Roberts A Murphy BR Subbarao K Nabel GJ A DNA vaccine induces SARS coronavirus neutralization and protective immunity in mice Nature 2004 428 561 564 10.1038/nature02463 15024391 PMC7095382

[B12] Thompson RE Jolliffe KA Payne RJ Total synthesis of microcin B17 via a fragment condensation approach Org Lett 2011 13 680 683 10.1021/ol102916b 21235262

[B13] Meister GE Roberts CG Berzofsky JA De Groot AS Two novel T cell epitope prediction algorithms based on MHC-binding motifs; comparison of predicted and published epitopes from Mycobacterium tuberculosis and HIV protein sequences Vaccin 1995 13 581 591 10.1016/0264-410X(94)00014-E 7483779

[B14] Buus S Lauemoller SL Worning P Kesmir C Frimurer T Corbet S Fomsgaard A Hilden J Holm A Brunak S Sensitive quantitative predictions of peptide-MHC binding by a 'query by committee' artificial neural network approach Tissue Antigens 2003 62 378 384 10.1034/j.1399-0039.2003.00112.x 14617044

[B15] Brusic V Bajic VB Petrovsky N Computational methods for prediction of T-cell epitopes–a framework for modelling, testing, and applications Methods 2004 34 436 443 10.1016/j.ymeth.2004.06.006 15542369

[B16] Peters B Sette A Generating quantitative models describing the sequence specificity of biological processes with the stabilized matrix method BMC Bioinform 2005 6 132 10.1186/1471-2105-6-132 PMC1173087 15927070

[B17] Peters B Tong W Sidney J Sette A Weng Z Examining the independent binding assumption for binding of peptide epitopes to MHC-I molecules Bioinform 2003 19 1765 1772 10.1093/bioinformatics/btg247 14512347

[B18] Doytchinova IA Guan P Flower DR EpiJen: a server for multistep T cell epitope prediction BMC Bioinform 2006 7 131 10.1186/1471-2105-7-131 PMC1421443 16533401

[B19] Bui HH Sidney J Peters B Sathiamurthy M Sinichi A Purton KA Mothe BR Chisari FV Watkins DI Sette A Automated generation and evaluation of specific MHC binding predictive tools: ARB matrix applications Immunogenet 2005 57 304 314 10.1007/s00251-005-0798-y 15868141

[B20] Peters B Bui HH Frankild S Nielson M Lundegaard C Kostem E Basch D Lamberth K Harndahl M Fleri W A community resource benchmarking predictions of peptide binding to MHC-I molecules PLoS Comput Biol 2006 2 e65 10.1371/journal.pcbi.0020065 16789818 PMC1475712

[B21] Wang P Sidney J Kim Y Sette A Lund O Nielsen M Peters B Peptide binding predictions for HLA DR DP and DQ molecules BMC Bioinform 2010 11 568 10.1186/1471-2105-11-568 PMC2998531 21092157

[B22] Parker KC Bednarek MA Coligan JE Scheme for ranking potential HLA-A2 binding peptides based on independent binding of individual peptide side-chains J Immunol 1994 152 163 175 8254189

[B23] Nielsen M Lund O NN-align. An artificial neural network-based alignment algorithm for MHC class II peptide binding prediction BMC Bioinform 2009 10 296 10.1186/1471-2105-10-296 PMC2753847 19765293

[B24] Wang B Chen H Jiang X Zhang M Wan T Li N Zhou X Wu Y Yang F Yu Y Identification of an HLA-A*0201-restricted CD8+ T-cell epitope SSp-1 of SARS-CoV spike protein Blood 2004 104 200 206 10.1182/blood-2003-11-4072 15016646 PMC8254376

[B25] Yang J James E Roti M Huston L Gebe JA Kwok WW Searching immunodominant epitopes prior to epidemic: HLA class II-restricted SARS-CoV spike protein epitopes in unexposed individuals Int Immunol 2009 21 63 71 19050106 10.1093/intimm/dxn124 PMC2638843

[B26] Zhi Y Kobinger GP Jordan H Suchma K Weiss SR Shen H Schumer G Gao G Boyer JL Crystal RG Identification of murine CD8 T cell epitopes in codon-optimized SARS-associated coronavirus spike protein Virol 2005 335 34 45 10.1016/j.virol.2005.01.050 PMC7111773 15823604

[B27] Boesteanu A Brehm M Mylin LM Christianson GJ Tevethia SS Roopenian DC Joyce S A molecular basis for how a single TCR interfaces multiple ligands J Immunol 1998 161 4719 4727 9794402

[B28] Robinson RA Lee DR Studies of tum- peptide analogs define an alternative anchor that can be utilized by Ld ligands lacking the consensus P2 anchor J Immunol 1996 156 4266 4273 8666797

[B29] Malik A Houghten R Corradin G Buus S Berzofsky JA Hoffman SL Identification of a nonameric H-2Kk-restricted CD8+ cytotoxic T lymphocyte epitope on the Plasmodium falciparum circumsporozoite protein Infect Immun 1995 63 1955 1959 7537251 10.1128/iai.63.5.1955-1959.1995 PMC173249

[B30] Mitaksov V Fremont DH Structural definition of the H-2Kd peptide-binding motif J Biol Chem 2006 281 10618 10625 10.1074/jbc.M510511200 16473882

[B31] Wells JA Additivity of mutational effects in proteins Biochem 1990 29 8509 8517 10.1021/bi00489a001 2271534

[B32] Arabshahi A Frey PA Standard free energy for the hydrolysis of adenylylated T4 DNA ligase and the apparent pKa of lysine 159 J Biol Chem 1999 274 8586 8588 10.1074/jbc.274.13.8586 10085093

[B33] Srinivasan KN Zhang GL Khan AM August JT Brusic V Prediction of class I T-cell epitopes: evidence of presence of immunological hot spots inside antigens Bioinform 2004 20 Suppl 1 i297 i302 10.1093/bioinformatics/bth943 PMC7110022 15262812

[B34] Soam SS Khan F Bhasker B Mishra BN Prediction of MHC class I binding peptides using probability distribution functions Bioinformation 2009 3 403 408 10.6026/97320630003403 19759816 PMC2732036

[B35] Lin HH Ray S Tongchusak S Reinherz EL Brusic V Evaluation of MHC class I peptide binding prediction servers: applications for vaccine research BMC Immunol 2008 9 8 10.1186/1471-2172-9-8 18366636 PMC2323361

[B36] Zhang GL Srinivasan KN Veeramani A August JT Brusic V PREDBALB/c: a system for the prediction of peptide binding to H2d molecules, a haplotype of the BALB/c mouse Nucleic Acids Res 2005 33 W180 W183 10.1093/nar/gki479 15980450 PMC1160239

[B37] Bolin DR Swain AL Sarabu R Berthel SJ Gillespie P Huby NJ Makofske R Orzechowski L Perrotta A Toth K Peptide and peptide mimetic inhibitors of antigen presentation by HLA-DR class II MHC molecules. Design, structure-activity relationships, and X-ray crystal structures J Med Chem 2000 43 2135 2148 10.1021/jm000034h 10841792

